# Neurologic Recovery at Discharge and Long-Term Survival After Cardiac Arrest

**DOI:** 10.1001/jamanetworkopen.2024.39196

**Published:** 2024-10-11

**Authors:** Emelie Dillenbeck, Leif Svensson, Araz Rawshani, Jacob Hollenberg, Mattias Ringh, Andreas Claesson, Akil Awad, Martin Jonsson, Per Nordberg

**Affiliations:** 1 Department of Clinical Science and Education, Center for Resuscitation Science, Södersjukhuset, Karolinska Institutet, Stockholm, Sweden; 2 Department of Medicine, Karolinska Institutet, Solna, Sweden; 3 Department of Molecular and Clinical Medicine, Institute of Medicine, University of Gothenburg, Gothenburg, Sweden; 4 Function Perioperative Medicine and Intensive Care, Karolinska University Hospital, Stockholm, Sweden; 5 Department of Physiology and Pharmacology, Karolinska Institutet, Stockholm, Sweden

## Abstract

**Importance:**

Brain injury is the leading cause of death following cardiac arrest and is associated with severe neurologic disabilities among survivors, with profound implications for patients and their families, as well as broader societal impacts. How these disabilities affect long-term survival is largely unknown.

**Objective:**

To investigate whether complete neurologic recovery at hospital discharge after cardiac arrest is associated with better long-term survival compared with moderate or severe neurologic disabilities.

**Design, Setting, and Participants:**

This cohort study used data from 4 mandatory national registers with structured and predefined data collection and nationwide coverage during a 10-year period in Sweden. Participants included adults who survived in-hospital cardiac arrest (IHCA) or out-of-hospital cardiac arrest (OHCA) beyond 30 days and who underwent predefined neurologic assessment conducted by health care professionals at hospital discharge using the Cerebral Performance Category (CPC) scale between January 2010 and December 2019. Patients were divided into 3 categories: complete recovery (CPC 1), moderate disabilities (CPC 2), and severe disabilities (CPC 3-4). Statistical analyses were performed in December 2023.

**Exposure:**

CPC score at hospital discharge.

**Main Outcomes and Measures:**

The primary outcome was long-term survival among patients with CPC 1 compared with those with CPC 2 or CPC 3 or 4.

**Results:**

A total of 9390 cardiac arrest survivors (median [IQR] age, 69 .0 [58.0-77.0] years; 6544 [69.7%] male) were included. The distribution of functional neurologic outcomes at discharge was 7374 patients (78.5%) with CPC 1, 1358 patients (14.5%) with CPC 2, and 658 patients (7.0%) with CPC 3 or 4. Survival proportions at 5 years were 73.8% (95% CI, 72.5%-75.0%) for patients with CPC 1, compared with 64.7% (95% CI, 62.4%-67.0%) for patients with CPC 2 and 54.2% (95% CI, 50.6%-57.8%) for patients with CPC 3 or 4. Compared with patients with CPC 1, there was significantly higher hazard of death for patients with CPC 2 (adjusted hazard ratio [aHR], 1.57 [95% CI, 1.40-1.75]) or CPC 3 or 4 (aHR, 2.46 [95% CI, 2.13-2.85]). Similar associations were seen in the OHCA and IHCA groups.

**Conclusions and Relevance:**

In this cohort study of patients with cardiac arrest who survived beyond 30 days, complete neurologic recovery, defined as CPC 1 at discharge, was associated with better long-term survival compared with neurologic disabilities at the same time point.

## Key Points

**Question:**

In patients with cardiac arrest, is complete neurologic recovery assessed at hospital discharge associated with better long-term survival compared with moderate or severe disabilities?

**Findings:**

This cohort study using 4 mandatory national Swedish registers and including 9390 survivors of cardiac arrest found that complete neurologic recovery at hospital discharge, defined as Cerebral Performance Category (CPC) 1 was significantly associated with better long-term survival (up to 8 years) compared with moderate (CPC 2) and more advanced disability (CPC 3-4).

**Meaning:**

This cohort study found that functional neurologic outcome at discharge was associated with long-term survival among survivors of cardiac arrest.

## Teaser

This cohort study examines the associations of neurologic recovery at hospital discharge after cardiac arrest with long-term survival among patients with in-hospital and out-of-hospital cardiac arrest in Sweden.

## Introduction

Post–cardiac arrest brain injury is a common cause of death in patients resuscitated from both out-of-hospital cardiac arrest (OHCA) and in-hospital cardiac arrest (IHCA).^1,2,3^ The mechanisms of injury consist of both global ischemia during cardiac arrest, as well as reperfusion injuries once circulation has been reestablished.^4^ The most important aim of post–cardiac arrest care is to minimize brain injury and thereby improve neurologic recovery.

Functional neurologic outcome in survivors of cardiac arrest has commonly been assessed by using the Cerebral Performance Category (CPC) scale, a 5-point scale ranging from CPC 1 (no or minimal neurologic disabilities), to CPC 5 (brain death).^5^ Despite considerable differences in neurologic function and the ability to resume to normal activity after the cardiac arrest between CPC 1 and CPC 2 (ie, moderate disabilities but independent in daily life), they are often both defined as favorable neurologic outcomes in cardiac arrest studies. Since 2018, the more detailed modified Rankin Scale^6^ has been the recommended measure of neurologic recovery in studies of cardiac arrest,^7^ although the CPC scale is still widely used.

The degree of neurologic recovery following cardiac arrest has a massive impact on the daily lives of patients and their families. Moreover, the broader societal impacts, measured as quality-of-life–adjusted life-years and health-related costs,^8,9,10,11^ including need for rehabilitation, are significant, although they have not been fully evaluated. In addition, there are indications that neurologic outcome may be a prognostic factor associated with long-term survival after cardiac arrest.^12,13,14,15,16,17^ However, most studies carried out to assess this potential association have been limited by small sample sizes, short follow-up time, or reliance on single-center populations. Although both CPC 1 and 2 are often considered favorable neurologic outcomes after cardiac arrest, there may be a difference in long-term survival between the 2 groups, with better a CPC score being associated with better long-term survival.^12,13,15,16^ To better assess this potential association, we used a large Swedish nationwide cohort with structurally collected neurologic outcome data, with the aim to investigate whether complete neurologic recovery, defined as CPC 1 at discharge, after IHCA and OHCA is associated with improved long-term survival compared with moderate or severe neurologic disabilities.

## Methods

This cohort study was approved by the Swedish Ethical Review Authority, and the requirement of informed consent was waived since this is not required in pseudoanonymized register-based research according to the Swedish law. This report follows the Strengthening the Reporting of Observational Studies in Epidemiology (STROBE) reporting guideline.

This is a nationwide, observational, register-based study including patients with cardiac arrest (OHCA and IHCA) between January 1, 2010, and December 31, 2019. The end of study follow-up was December 31, 2020. The study was conducted in Sweden, with a population of 10.3 million inhabitants in 2019.^18^ Data were obtained from the Swedish Register for Cardiopulmonary Resuscitation (SRCR), the Swedish Cause of Death Register, the National Patient Registry, and Statistics Sweden.

### Patients

Participants included patients with IHCA or OHCA, with a personal identity number, reported to the SRCR. Patients aged younger than 18 years, who died within 30 days of cardiac arrest, with OHCA not treated by the emergency medical services (EMS), or with no registered CPC score at discharge were excluded.

### Data Sources

All residents in Sweden have a unique personal identity number. This allows for linking of data from the different registers.

#### SRCR

The SRCR is a national quality register with nationwide data on both OHCA and IHCA. All patients with cardiac arrest for whom attempts at resuscitation have been made are included, and data are reported in accordance with Utstein guidelines.^19,20^ All EMS systems report to the register, and in 2010, 74% of Swedish hospitals reported data on IHCA to the SRCR, increasing to 98% of hospitals in 2019.^21,22^ Prehospital data are registered by EMS personnel in close connection to the event. In-hospital data are registered by a local cardiopulmonary resuscitation (CPR) coordinator who is a specialized nurse. CPC scores at discharge are determined by the specially trained CPR coordinator from hospital records. Patients alive after 3 months are informed that they are included in the register and that they can withdraw their data from the register at any time. The register has been validated and thoroughly described elsewhere.^23^

#### Swedish Cause of Death Register and National Patient Register

The Swedish Cause of Death Register is a national register of all deaths in Sweden since 1952. It is based on medical death certificates, which must be made by a physician and sent to the National Board of Health and Welfare within 3 weeks of the death.^24^ The National Patient Register contains *International Statistical Classification of Diseases and Related Health Problems, Tenth Revision *(*ICD-10*) codes on all in-patient care in Sweden since 1987, and *ICD-10* codes on outpatient visits to hospitals and specialist clinics since 2001.^25^ Both registers are maintained by the Swedish National Board of Health and Welfare. In our study, data on date of death were collected from the Swedish Cause of Death Register, and diagnoses of comorbidities up to 30 days after cardiac arrest were collected from the National Patient Register.

#### Statistics Sweden

Statistics Sweden is responsible for the official statistics of Sweden.^26^ Data on disposable income (mean disposable income during 10 years before cardiac arrest) and educational level (highest attained educational level) were obtained from the Longitudinal Integrated Database for Health Insurance and Labor Market Studies,^27^ in which data are available from 1990 onward for the Swedish population. Data on birth region were obtained from the Total Population Register, which, since 1968, contains data on birth, death, name change, marital status, family relationships, and migration.^28^

### Exposure

The exposure was functional neurologic outcome at discharge, defined by the CPC score, in which CPC 1 indicates no or minimal neurological injury; CPC 2, moderate neurological disability but sufficient cerebral function to live independently and work in a sheltered environment CPC 3, severe neurological disability (conscious but dependent on others); CPC 4, persistent vegetative state; and CPC 5, equivalent to death.^5^ In this study, patients with CPC scores of 3 and 4 were analyzed as 1 group, due to limited numbers in each category.

### Outcome Measures

The primary outcome was survival during the follow-up period for all patients with cardiac arrest. Secondary outcomes were survival during the same period in the subgroups of OHCA and IHCA.

### Statistical Analysis

For continuous variables, descriptive statistics are presented as medians and IQRs. Categorial data are presented as frequencies and percentages. Differences in baseline characteristics were addressed by assessing standardized mean differences.

Cox proportional hazards regression models were used to estimate survival associated with CPC after adjusting for age, sex, initial rhythm, witnessed cardiac arrest, bystander CPR, year of cardiac arrest, OHCA or IHCA, birth region, income, and comorbidities (ie, ischemic heart disease [IHD], heart failure, chronic obstructive pulmonary disease [COPD], diabetes, cancer, kidney failure, and dementia). For subanalyses, the population was divided into OHCA and IHCA and the data were analyzed separately. Subanalyses were also carried out with the population divided into 2 groups according to age at cardiac arrest: 65 years or younger and older than 65 years. This was done because expected long-term survival differs with age, and a cutoff at 65 years of age was chosen since it was the standard retirement age in Sweden during the study period. To visualize the results, we plotted adjusted survival curves using the direct method (from the Cox model), together with the adjusted hazard ratios (aHRs) and 95% CIs. The adjusted curves were obtained by using the adjustedCurves package in R software version 4.2.2 (R Project for Statistical Computing).^29^ In addition, Cox proportional hazards regression models were used to estimate survival associated with CPC score in the subgroups with shockable and nonshockable rhythms, with witnessed cardiac arrest and shockable rhythm, and with and without a diagnosis of IHD.

To compare the association between CPC score at discharge and long-term survival with other factors possibly associated with long-term survival after cardiac arrest, we performed multivariate regression analyses for all patients and for the subgroups of OHCA and IHCA. Adjustments were made for age, sex, year of cardiac arrest, and diagnosis of heart failure, cancer, and diabetes.

Statistical analyses were performed in December 2023 using R software. *P* values were 2-sided, and statistical significance was set at *P* ≤ .05.

## Results

### Patients and Baseline Characteristics

**Figure 1.**
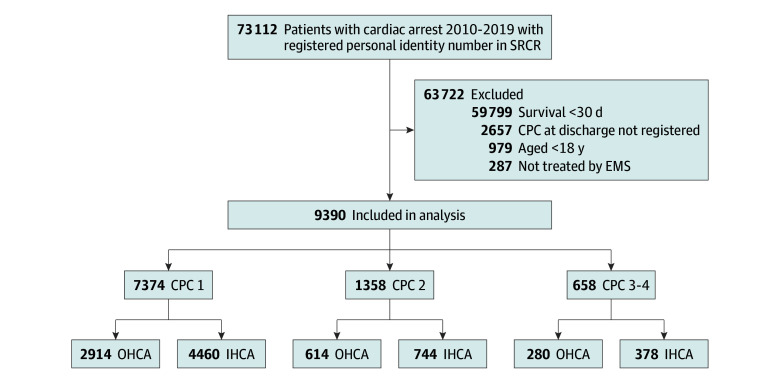
Flowchart of Included Patients CPC indicates Cerebral Performance Category; EMS, emergency medical services; IHCA, in-hospital cardiac arrest; OHCA, out-of-hospital cardiac arrest. CPC 1 indicates no or minimal neurological injury; CPC 2, moderate neurological disability but sufficient cerebral function to live independently and work in a sheltered environment; CPC 3, severe neurological disability (conscious but dependent on others); CPC 4, persistent vegetative state.

A total of 73 112 patients with IHCA or OHCA were registered in the SRCR with a personal identity number between January 1, 2010, and December 31, 2019. After excluding patients not treated by the EMS, those younger than 18 years, patients who did not survive 30 days, and patients without a registered CPC score at discharge, 9390 patients (median [IQR] age, 69.0 [58.0-77.0] years, 6544 [69.7%] male) were included in the analysis. A total of 5214 patients (65.3%) had a shockable rhythm. At discharge, 7374 patients (78.5%) had CPC 1, 1358 patients (14.5%) had CPC 2, and 658 patients (7.0%) had CPC 3 or 4 (Figure 1). There were 3808 patients with OHCA and 5582 patients with IHCA, and the proportion of CPC scores were similar in these 2 subgroups.

The Table shows patients characteristics according to CPC score. The proportion of male or female sex did not differ between groups, nor did region of birth. Patients with CPC 2 were the oldest, with median (IQR) age 72.0 (62.3-81.0) years. There was a lower proportion of shockable rhythms the higher the CPC score. There were more patients with a disposable income in the fourth quartile among those with a CPC score of 1. Diagnoses of cerebral infarction and dementia were more common in the CPC 2 and CPC 3 or 4 groups, and patients with CPC 3 or 4 showed a lower proportion of IHD. PCI was more commonly performed the higher the CPC score. Patient characteristics by OHCA and IHCA are presented in eTable 1 and eTable 2 in Supplement 1. Baseline characteristics for eligible patients excluded from analysis because of a missing CPC score at discharge were similar (eTable 3 in Supplement 1).

**Table.** Baseline Characteristics of All Cardiac Arrests

Characteristic	Participants, No. (%)			SMD (95%CI)		Missing, %
	CPC 1 (n = 7374)	CPC 2 (n = 1358)	CPC 3-4 (n = 658)	CPC 1 vs CPC 2	CPC 1 vs CPC 3-4	
Location						
IHCA	2914 (39.5)	614 (45.2)	280 (42.6)	0.12 (0.06-0.17)	0.06 (-0.02-0.14)	0.0
OHCA	4460 (60.5)	744 (54.8)	378 (57.4)			
Sex						
Female	2192 (29.7)	441 (32.5)	211 (32.1)	0.06 (0.00 to 0.12)	0.05 (−0.03 to 0.13)	<0.1
Male	5181 (70.3)	917 (67.5)	446 (67.9)			
Age, median (IQR), y	68 (58.0 to 76)	72 (62.3 to 81)	69 (56.0 to 78)	−0.28 (−0.34 to −0.22)	−0.01 (−0.09 to 0.07)	0.0
Witnessed cardiac arrest	6761 (92.9)	1214 (90.9)	578 (89.3)	0.07 (0.01 to 0.13)	0.13 (0.04 to 0.21)	1.4
Shockable rhythm^a^	4276 (67.7)	688 (60.4)	250 (47.6)	0.15 (0.09 to 0.22)	0.41 (0.32 to 0.50)	15
PCI^b^	1140 (39.6)	189 (34.7)	74 (26.4)	0.10 (0.01 to 0.19)	0.28 (0.16 to 0.41)	61
CABG^b^	83 (2.9)	15 (2.8)	5 (1.8)	0.01 (−0.09 to 0.10)	0.07 (−0.05 to 0.20)	61
ICD^b^	519 (19.1)	123 (22.7)	46 (17.4)	−0.09 (−0.18 to 0.00)	0.04 (−0.08 to 0.17)	62
TTM (32-26 °C)^b^	1003 (13.9)	326 (24.8)	205 (32.3)	−0.28 (−0.34 to −0.22)	−0.45 (−0.53 to −0.37)	2.4
Birth region^c^						
Nordic countries	6559 (92.2)	1231 (93.8)	577 (91.7)	0.06 (0.06 to 0.12)	0.02 (−0.06 to 0.10)	3.6
Other European countries	203 (2.9)	28 (2.1)	18 (2.9)			
Other	349 (4.9)	54 (4.1)	34 (5.4)			
Income, quartile^d^						
First (lowest)	1724 (23.5)	333 (24.6)	185 (28.5)	0.15 (0.09 to 0.21)	0.20 (0.12 to 0.28)	0.5
Second	1763 (24.0)	393 (29.1)	179 (27.6)			
Third	1866 (25.4)	335 (24.8)	158 (24.3)			
Fourth (higest)	1991 (27.1)	291 (21.5)	127 (19.6)			
Educational level						
Primary	2470 (33.6)	494 (36.6)	243 (37.4)	0.10 (0.04 to 0.15)	0.12 (0.04 to 0.20)	0.5
Secondary	3091 (42.1)	565 (41.8)	246 (37.9)			
Postsecondary ≤2 y	775 (10.6)	109 (8.1)	57 (8.8)			
Postsecondary ≥3 y	1008 (13.7)	183 (13.5)	103 (15.9)			
Comorbidities^e^						
IHD	2869 (38.9)	518 (38.1)	198 (30.1)	0.02 (−0.04 to 0.07)	0.19 (0.11 to 0.27)	0.0
Heart failure	1435 (19.5)	363 (26.7)	135 (20.5)	−0.17 (−0.23 to −0.12)	−0.03 (−0.11 to 0.05)	0.0
COPD	515 (7.0)	113 (8.3)	50 (7.6)	−0.05 (−0.11 to 0.01)	−0.02 (−0.10 to 0.06)	0.0
Cerebral infarction	457 (6.2)	197 (14.5)	100 (15.2)	−0.28 (−0.33 to −0.22)	−0.29 (−0.37 to −0.21)	0.0
Diabetes	1299 (17.6)	295 (21.7)	123 (18.7)	−0.10 (−0.16 to −0.05)	−0.03 (−0.11 to 0.05)	0.0
Hypertension	3174 (43.0)	689 (50.7)	305 (46.4)	−0.15 (−0.21 to −0.10)	−0.07 (−0.15 to 0.01)	0.0
Cancer	1371 (18.6)	300 (22.1)	120 (18.2)	−0.09 (−0.14 to −0.03)	0.01 (−0.07 to 0.09)	0.0
Kidney failure	584 (7.9)	152 (11.2)	65 (9.9)	−0.11 (−0.17 to −0.05)	−0.07 (−0.15 to 0.01)	0.0
Dementia	126 (1.7)	95 (7.0)	68 (10.3)	−0.26 (−0.32 to −0.20)	−0.37 (−0.45 to −0.29)	0.0

Abbreviations: CABG, coronary artery bypass graft; COPD, chronic obstructive pulmonary disease; CPC, Cerebral Performance Category; ICD, implantable cardioverter defibrillator; IHCA, in-hospital cardiac arrest; IHD, ischemic heart disease; OHCA, out-of-hospital cardiac arrest; PCI, percutaneous coronary intervention; SMD, standardized mean difference; TTM, targeted temperature management.

^a^ Ventricular fibrillation or pulseless ventricular tachycardia.

^b^ During hospitalization for cardiac arrest.

^c^ Nordic countries include Sweden, Norway, Denmark and Iceland. Others include countries in Africa, North America, South America, and Asia.

^d^ Mean disposable income 10 years before cardiac arrest.

^e^ Diagnosed up to 30 days after cardiac arrest.

### Outcomes

**Figure 2.**
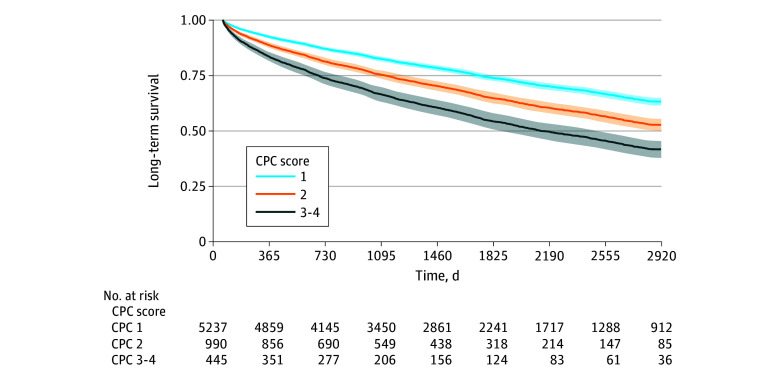
Adjusted Survival Proportions by Cerebral Performance Category (CPC) at 1, 3, 5, and 8 Years of Follow-Up CPC 1 indicates no or minimal neurological injury; CPC 2, moderate neurological disability but sufficient cerebral function to live independently and work in a sheltered environment; CPC 3, severe neurological disability (conscious but dependent on others); CPC 4, persistent vegetative state.

During the study period, there were 3102 deaths in our population. The median (range) time from cardiac arrest to death or the end of study follow-up was 1452 (61-4046) days, or approximately 4 years (2 months to 11 years). A more favorable CPC score at discharge was associated with better long-term survival (Figure 2).

Adjusted survival proportions at 1 year were 92.3% (95% CI, 91.7%-93.0%) for patients in the CPC 1 group, compared with 88.6% (95% CI, 87.4%-89.7%) for those in the CPC 2 group and 83.4% (95% CI, 81.3%-85.5%) for those in the CPC 3 or 4 group. Adjusted survival proportions at 5 years were 73.8% (95% CI, 72.5%-75.0%) for CPC 1, 64.7% (95% CI, 62.4%-67.0%) for CPC 2, and 54.2% (95% CI, 50.6%-57.8%) for CPC 3 or 4. At 8 years, the adjusted survival proportions were 63.1% (95% CI, 61.4%-64.8%) for CPC 1, 52.6% (95% CI, 49.9%-55.3%) for CPC 2, and 41.6% (95% CI, 37.8%-45.3%) for CPC 3 or 4.

**Figure 3.**
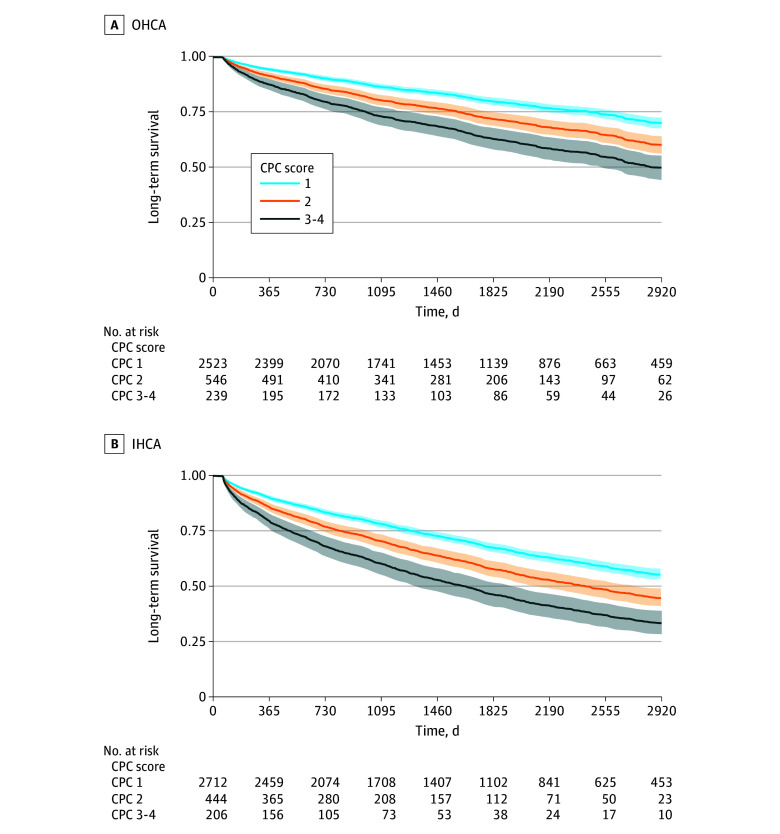
Adjusted Survival Proportions by Cerebral Performance Category (CPC) in the Out-of-Hospital Cardiac Arrest (OHCA) and In-Hospital Cardiac Arrest (IHCA) Groups CPC 1 indicates no or minimal neurological injury; CPC 2, moderate neurological disability but sufficient cerebral function to live independently and work in a sheltered environment; CPC 3, severe neurological disability (conscious but dependent on others); CPC 4, persistent vegetative state.

Compared with the CPC 1 group, hazard for death increased incrementally, with higher CPC score associated with greater hazard (CPC 2: aHR, 1.57 [95% CI, 1.40-1.75]; CPC 3-4: aHR, 2.46 [95% CI, 2.13-2.85]). Similar associations were seen in the OHCA (CPC 2: aHR, 1.61 [95% CI, 1.36-1.91]; CPC 3-4: aHR, 2.53 [95% CI, 2.03-3.15]) and IHCA (CPC 2: aHR, 1.51 [95% CI, 1.30-1.77]; CPC 3-4: aHR, 2.38 [95% CI, 1.96-2.90]) groups (Figure 3) and in patients aged 65 years or younger and older than 65 years (eFigure 1 in Supplement 1). The subgroups of patients with and without a diagnosis of IHD, as well as the subgroups with shockable and nonshockable rhythms and witnessed cardiac arrest with shockable rhythm also showed the same associations (eTable 4 in Supplement 1). Kaplan-Meier curves for all patients and for the OHCA and IHCA groups are presented in eFigure 2 and eFigure 3 in Supplement 1).

**Figure 4.**
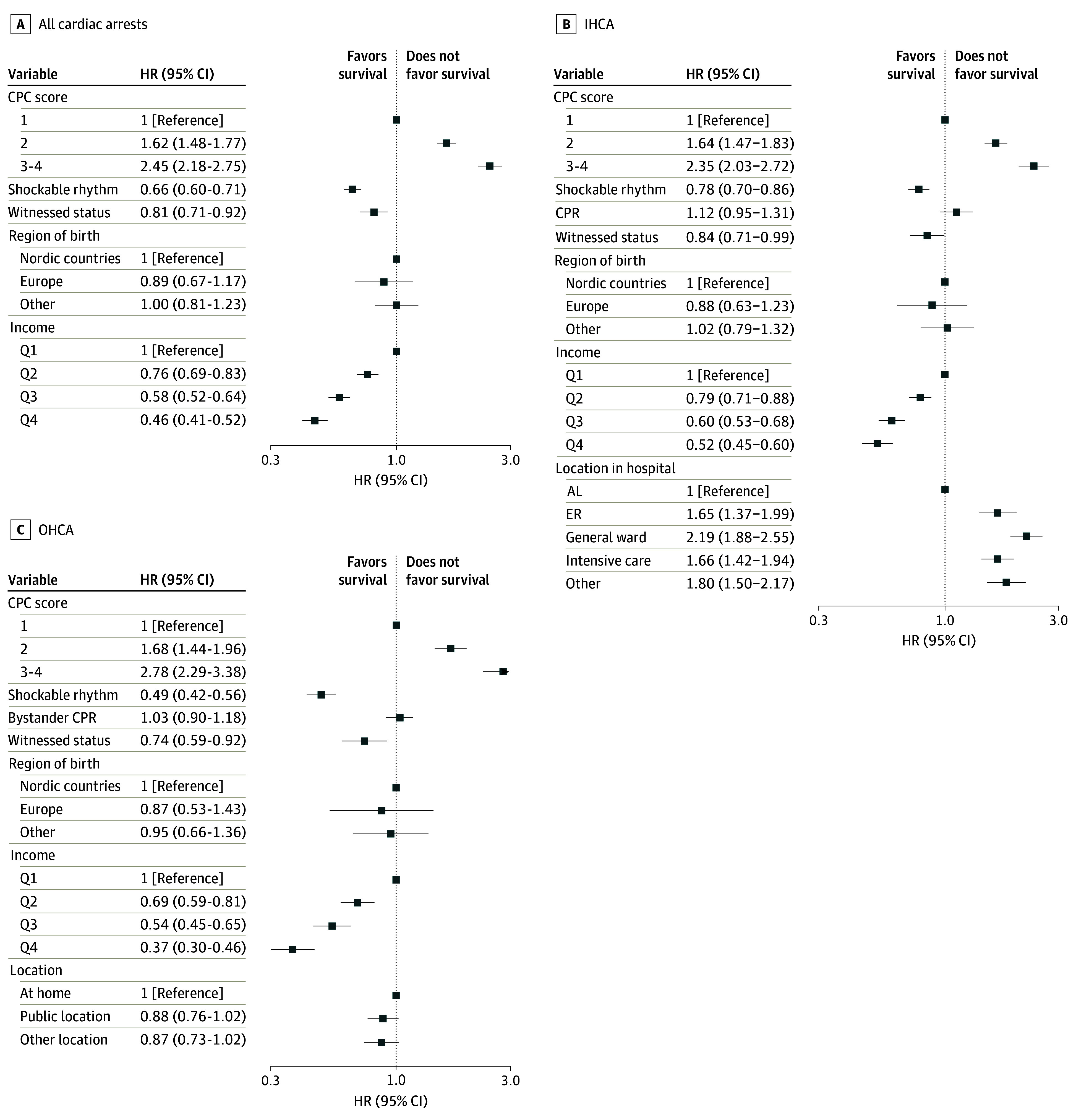
Factors Associated With Long-Term Survival Income is mean disposable income 10 years before cardiac arrest in quartiles (Q) with Q1 indicating lowest and Q4, highest. AL indicates angiography laboratory; CPC, cerebral performance category; CPR, cardiopulmonary resuscitation; ER; emergency department; HR, hazard ratio. CPC 1 indicates no or minimal neurological injury; CPC 2, moderate neurological disability but sufficient cerebral function to live independently and work in a sheltered environment; CPC 3, severe neurological disability (conscious but dependent on others); CPC 4, persistent vegetative state.

CPC scores, together with other factors, and their association with long-term survival are shown in Figure 4. For all patients, as well as in subgroups of IHCA and OHCA, CPC greater than 1 at discharge was associated with reduced long-term survival, while shockable rhythm, witnessed cardiac arrest, and high income were associated with better long-term survival. For IHCA, cardiac arrest at all places other than the catheterization laboratory was associated with lower long-term survival, while cardiac arrest location in the OHCA population was not significantly associated with long-term survival.

## Discussion

This nationwide cohort study using mandatory high-quality registers and including 30-day survivors from cardiac arrest in Sweden found that complete neurologic recovery at hospital discharge, defined as CPC 1, was significantly associated with improved long-term survival compared with moderate neurologic disabilities (CPC 2) and severe disabilities or coma (CPC 3 to 4) at the same time point. Similar associations were seen in the OHCA and IHCA subgroups and in patients aged 65 years or younger and older than 65 years.

Our findings that neurologic status at discharge was associated with long-term prognosis are consistent with those of previous smaller studies on both OHCA^12,13,14,15,17^ and IHCA^15,16^ and suggest that the CPC score at discharge might be a predictor of long-term survival in patients surviving cardiac arrest beyond 30 days, although further prospective studies are needed to confirm this. Our findings might be useful for clinicians in their discussions with surviving patients and their relatives to give information regarding potential long-term outcomes. The difference in survival between patients assessed as CPC 1 and CPC 2 at discharge is especially interesting, since these 2 categories are often both considered favorable outcomes in studies on cardiac arrest. Our results further support the importance of postresuscitation care aiming to restore neurologic function, as this potentially leads to extended years of life. One might even argue that only CPC 1, or corresponding levels of other measures of neurologic outcome, should be considered a favorable outcome in studies presenting outcomes after cardiac arrest.

The difference in survival between the CPC groups may have several explanations. Functional neurologic recovery could, for example, affect the level of physical activity, adherence to medical treatments, and the ability to seek health care. However, the difference in survival might also be explained by a higher burden of comorbidities in patients with higher CPC scores. Some studies have shown that better Charlson Comorbidity Index (CCI)^30^ is associated with better functional neurologic outcome after OHCA,^31^ while others have not.^32^ We did not have full CCI data for our patients, but adjusting for many of the comorbidities included in the CCI in our analysis did not markedly affect the association between neurologic outcome and long-term survival. Whether there are differences in follow-up treatment or treatment of other comorbidities among the groups, which might affect long-term survival, is an important question for further studies.

Aside from the more expected associations with initial shockable rhythm and cardiac arrest location, we also observed an association between high income and long-term survival. Previous studies on patients with OHCA have found that socioeconomic status is associated with bystander CPR and short-term survival,^33,34,35^ and, similar to our results, reported that high income is associated with better long-term survival.^36^ Socioeconomic status affects lifestyle factors, such as smoking, dietary habits, physical activity, and body mass index, and thereby long-term survival.^37,38^ We do not have data on lifestyle factors, but by adjusting for individual-level income and birth region we have to some extent reduced the effect of this potential confounder.

In our subgroup analysis, the IHCA group showed reduced long-term survival compared with patients with OHCA in all CPC groups. In contrast, previous studies have reported higher 30-day and 1-year survival rates in patients with IHCA vs patients with OHCA^39,40,41^ or no difference in long-term survival at longer than 10 years.^42^ Compared with patients with OHCA, patients with IHCA generally have a lower proportion of cardiac causes,^43^ and the fact that they are already hospitalized at the time of cardiac arrest suggests a higher burden of acute illness and comorbidities, but they also have proximity to high-quality advanced cardiovascular life support and post–cardiac arrest care, which could increase the survival rate. However, long-term survival could be more affected by age and comorbidities than survival at 30 days or 1 year. Our IHCA population was older than the OHCA population and had higher proportions of comorbidities, and more than one-third of our IHCA population was already in an intensive care unit when cardiac arrest occurred. This might explain the lower rate of long-term survival in the IHCA cohort.

The major strength of this study is the large sample size, covering nearly all cardiac arrests in Sweden during the study period, as well as the long follow-up period. The strong association between CPC score at discharge and long-term survival was consistent in the OHCA and IHCA subgroups and both age groups, as well as with previous, smaller studies, which further adds to the strengths of this study.

### Limitations

Our study has several limitations. First, the length of follow-up varied in the cohort as a result of inclusion of patients up to 2019 and follow-up to the end of 2020, which affects the generalizability of the findings. Second, 2657 patients eligible for the study were missing CPC scores at discharge and were therefore excluded from the analysis, which could have introduced selection bias. Third, since data were collected from a register, determination of CPC scores was carried out by different persons and not cross-checked, which might have affected the accuracy of scoring. That CPC scoring was made from hospital records and not clinical visits also might have affected the scoring and induced potential bias. There also might have been postdischarge shifts in CPC levels. Fourth, during the studied period, changes in guidelines for targeted temperature management occurred, which meant that postresuscitation care differed between patients. There might also have been other changes in medical management during the inclusion period. This has not been accounted for other than by adjusting for year of cardiac arrest in the survival analysis. Fifth, the observational design of the study cannot exclude the potential of residual confounding and the results should be interpreted as such.

## Conclusions

In this nationwide cohort study in Sweden, complete neurologic recovery at hospital discharge among 30-day survivors after IHCA and OHCA was associated with better long-term survival compared with moderate or severe neurologic disabilities at the same time point. Similar associations were seen in the OHCA and IHCA subgroups.

## Notes

Supplement 1. **eTable 1. **Baseline Characteristics of the OHCA Population **eTable 2.** Baseline Characteristics of the IHCA Population **eTable 3.** Baseline Characteristics of Eligible Patients With Missing CPC-Score at Discharge **eTable 4.** Associations Between CPC Score and Long-Term Survival in the Subpopulations With and Without a Diagnosis of IHD, With Shockable and Nonshockable Rhythms and Witnessed Cardiac Arrests With Shockable Rhythm **eFigure 1.** Adjusted Survival Proportions by Cerebral Performance Category (CPC) for Patients Aged ≤65 Years and >65 Years **eFigure 2.** Kaplan-Meier Plot for All Patients **eFigure 3.** Kaplan-Meier Plot for the Subgroups of Patients With Out-of-Hospital Cardiac Arrest (OHCA) and In-Hospital Cardiac Arrest (IHCA)

Supplement 2.
Data Sharing Statement

## References

[1] Laver S, Farrow C, Turner D, Nolan J. Mode of death after admission to an intensive care unit following cardiac arrest. Intensive Care Med. 2004;30(11):2126-2128. doi:10.1007/s00134-004-2425-z 15365608

[2] Witten L, Gardner R, Holmberg MJ, . Reasons for death in patients successfully resuscitated from out-of-hospital and in-hospital cardiac arrest. Resuscitation. 2019;136:93-99. doi:10.1016/j.resuscitation.2019.01.031 30710595 PMC6476296

[3] Lemiale V, Dumas F, Mongardon N, . Intensive care unit mortality after cardiac arrest: the relative contribution of shock and brain injury in a large cohort. Intensive Care Med. 2013;39(11):1972-1980. doi:10.1007/s00134-013-3043-4 23942856

[4] Sekhon MS, Ainslie PN, Griesdale DE. Clinical pathophysiology of hypoxic ischemic brain injury after cardiac arrest: a “two-hit” model. Crit Care. 2017;21(1):90. doi:10.1186/s13054-017-1670-9 28403909 PMC5390465

[5] Jennett B, Bond M. Assessment of outcome after severe brain damage. Lancet. 1975;1(7905):480-484. doi:10.1016/s0140-6736(75)92830-5 46957

[6] Rankin J. Cerebral vascular accidents in patients over the age of 60—II: prognosis. Scott Med J. 1957;2(5):200-215. doi:10.1177/003693305700200504 13432835

[7] Haywood K, Whitehead L, Nadkarni VM, ; COSCA Collaborators. COSCA (Core Outcome Set for Cardiac Arrest) in adults: an advisory statement from the International Liaison Committee on Resuscitation. Resuscitation. 2018;127:147-163. doi:10.1016/j.resuscitation.2018.03.022 29706235

[8] Chirikov VV, Corman S, Qiao Y, Huang X. Clinical and economic burden of out-of-hospital cardiac arrest in US commercial insurance population (2014 to 2019). Am J Cardiol. 2022;169:42-50. doi:10.1016/j.amjcard.2021.12.038 35063266

[9] Chan PS, Nallamothu BK, Krumholz HM, ; American Heart Association’s Get With the Guidelines-Resuscitation Investigators. Readmission rates and long-term hospital costs among survivors of an in-hospital cardiac arrest. Circ Cardiovasc Qual Outcomes. 2014;7(6):889-895. doi:10.1161/circoutcomes.114.000925 25351479 PMC4241155

[10] Geri G, Dumas F, Bonnetain F, . Predictors of long-term functional outcome and health-related quality of life after out-of-hospital cardiac arrest. Resuscitation. 2017;113:77-82. doi:10.1016/j.resuscitation.2017.01.028 28202421

[11] Efendijev I, Folger D, Raj R, . Outcomes and healthcare-associated costs one year after intensive care-treated cardiac arrest. Resuscitation. 2018;131:128-134. doi:10.1016/j.resuscitation.2018.06.028 29958958

[12] Phelps R, Dumas F, Maynard C, Silver J, Rea T. Cerebral Performance Category and long-term prognosis following out-of-hospital cardiac arrest. Crit Care Med. 2013;41(5):1252-1257. doi:10.1097/CCM.0b013e31827ca975 23388519

[13] Chocron R, Fahrenbruch C, Yin L, . Association between functional status at hospital discharge and long-term survival after out-of-hospital-cardiac-arrest. Resuscitation. 2021;164:30-37. doi:10.1016/j.resuscitation.2021.04.031 33965475

[14] Pachys G, Kaufman N, Bdolah-Abram T, Kark JD, Einav S. Predictors of long-term survival after out-of-hospital cardiac arrest: the impact of Activities of Daily Living and Cerebral Performance Category scores. Resuscitation. 2014;85(8):1052-1058. doi:10.1016/j.resuscitation.2014.03.312 24727137

[15] Hsu CH, Li J, Cinousis MJ, . Cerebral performance category at hospital discharge predicts long-term survival of cardiac arrest survivors receiving targeted temperature management*. Crit Care Med. 2014;42(12):2575-2581. doi:10.1097/ccm.0000000000000547 25072759 PMC4236246

[16] Chan PS, Nallamothu BK, Krumholz HM, ; American Heart Association Get with the Guidelines–Resuscitation Investigators. Long-term outcomes in elderly survivors of in-hospital cardiac arrest. N Engl J Med. 2013;368(11):1019-1026. doi:10.1056/NEJMoa1200657 23484828 PMC3652256

[17] Chan PS, McNally B, Nallamothu BK, . Long-term outcomes among elderly survivors of out-of-hospital cardiac arrest. J Am Heart Assoc. 2016;5(3):e002924. doi:10.1161/jaha.115.002924 27068632 PMC4943267

[18] Statistics Sweden. Folkmängden efter region, civilstånd, ålder och kön: År 1968-2021. Accessed December 9, 2022. https://www.statistikdatabasen.scb.se/pxweb/sv/ssd/START__BE__BE0101__BE0101A/BefolkningNy/

[19] Langhelle A, Nolan J, Herlitz J, ; 2003 Utstein Consensus Symposium. Recommended guidelines for reviewing, reporting, and conducting research on post-resuscitation care: the Utstein style. Resuscitation. 2005;66(3):271-283. doi:10.1016/j.resuscitation.2005.06.005 16129543

[20] Perkins GD, Jacobs IG, Nadkarni VM, ; Utstein Collaborators. Cardiac arrest and cardiopulmonary resuscitation outcome reports: update of the Utstein Resuscitation Registry Templates for Out-of-Hospital Cardiac Arrest: a statement for healthcare professionals from a task force of the International Liaison Committee on Resuscitation (American Heart Association, European Resuscitation Council, Australian and New Zealand Council on Resuscitation, Heart and Stroke Foundation of Canada, InterAmerican Heart Foundation, Resuscitation Council of Southern Africa, Resuscitation Council of Asia); and the American Heart Association Emergency Cardiovascular Care Committee and the Council on Cardiopulmonary, Critical Care, Perioperative and Resuscitation. Circulation. 2015;132(13):1286-1300. doi:10.1161/cir.0000000000000144 25391522

[21] Svenska hjärt-lungräddningsregistret (Swedish Register for Cardiopulmonary Rescusitation). Nationellt register för hjärtstopp årsrapport 2010. Accessed December 9, 2022. https://registercentrum.blob.core.windows.net/shlrsjh/r/-rsrapport-2010-BkRj7ufG-.pdf

[22] Svenska hjärt-lungräddningsregistret (Swedish Register for Cardiopulmonary Rescusitation). Årsrapport för år 2019. Accessed December 9, 2022. https://arsrapporter.registercentrum.se/shlr/20201103/

[23] Strömsöe A, Svensson L, Axelsson ÅB, Göransson K, Todorova L, Herlitz J. Validity of reported data in the Swedish Cardiac Arrest Register in selected parts in Sweden. Resuscitation. 2013;84(7):952-956. doi:10.1016/j.resuscitation.2012.12.026 23313425

[24] Brooke HL, Talbäck M, Hörnblad J, . The Swedish cause of death register. Eur J Epidemiol. 2017;32(9):765-773. doi:10.1007/s10654-017-0316-1 28983736 PMC5662659

[25] Swedish National Board of Health and Welfare. National Patient Register: Stockholm 2018. Updated February 22, 2018. Accessed April 24, 2023. https://www.socialstyrelsen.se/en/statistics-and-data/registers/national-patient-register/

[26] Statistics Sweden. Accessed April 24, 2023. https://www.scb.se/en/finding-statistics/

[27] Ludvigsson JF, Svedberg P, Olén O, Bruze G, Neovius M. The longitudinal integrated database for health insurance and labour market studies (LISA) and its use in medical research. Eur J Epidemiol. 2019;34(4):423-437. doi:10.1007/s10654-019-00511-8 30929112 PMC6451717

[28] Ludvigsson JF, Almqvist C, Bonamy AK, . Registers of the Swedish total population and their use in medical research. Eur J Epidemiol. 2016;31(2):125-136. doi:10.1007/s10654-016-0117-y 26769609

[29] Denz R, Klaaßen-Mielke R, Timmesfeld N. A comparison of different methods to adjust survival curves for confounders. Stat Med. 2023;42(10):1461-1479. doi:10.1002/sim.9681 36748630

[30] Charlson ME, Pompei P, Ales KL, MacKenzie CR. A new method of classifying prognostic comorbidity in longitudinal studies: development and validation. J Chronic Dis. 1987;40(5):373-383. 3558716 10.1016/0021-9681(87)90171-8

[31] Dumas F, Blackwood J, White L, . The relationship between chronic health conditions and outcome following out-of-hospital ventricular fibrillation cardiac arrest. Resuscitation. 2017;120:71-76. doi:10.1016/j.resuscitation.2017.08.239 28860011

[32] Beesems SG, Blom MT, van der Pas MH, . Comorbidity and favorable neurologic outcome after out-of-hospital cardiac arrest in patients of 70 years and older. Resuscitation. 2015;94:33-39. doi:10.1016/j.resuscitation.2015.06.017 26116780

[33] van Nieuwenhuizen BP, Oving I, Kunst AE, . Socio-economic differences in incidence, bystander cardiopulmonary resuscitation and survival from out-of-hospital cardiac arrest: a systematic review. Resuscitation. 2019;141:44-62. doi:10.1016/j.resuscitation.2019.05.018 31199944

[34] Chamberlain RC, Barnetson C, Clegg GR, Halbesma N. Association of measures of socioeconomic position with survival following out-of-hospital cardiac arrest: A systematic review. Resuscitation. 2020;157:49-59. doi:10.1016/j.resuscitation.2020.09.025 33010372

[35] Jonsson M, Härkönen J, Ljungman P, . Inequalities in income and education are associated with survival differences after out-of-hospital cardiac arrest: nationwide observational study. Circulation. 2021;144(24):1915-1925. doi:10.1161/circulationaha.121.056012 34767462 PMC8663522

[36] Møller S, Wissenberg M, Søndergaard K, . Long-term outcomes after out-of-hospital cardiac arrest in relation to socioeconomic status. Resuscitation. 2021;167:336-344. doi:10.1016/j.resuscitation.2021.07.015 34302925

[37] Stringhini S, Sabia S, Shipley M, . Association of socioeconomic position with health behaviors and mortality. JAMA. 2010;303(12):1159-1166. doi:10.1001/jama.2010.297 20332401 PMC2918905

[38] Dugravot A, Fayosse A, Dumurgier J, . Social inequalities in multimorbidity, frailty, disability, and transitions to mortality: a 24-year follow-up of the Whitehall II cohort study. Lancet Public Health. 2020;5(1):e42-e50. doi:10.1016/s2468-2667(19)30226-9 31837974 PMC7098476

[39] Andersson A, Arctaedius I, Cronberg T, . In-hospital versus out-of-hospital cardiac arrest: characteristics and outcomes in patients admitted to intensive care after return of spontaneous circulation. Resuscitation. 2022;176:1-8. doi:10.1016/j.resuscitation.2022.04.023 35490935

[40] Høybye M, Stankovic N, Holmberg M, Christensen HC, Granfeldt A, Andersen LW. In-hospital vs. out-of-hospital cardiac arrest: patient characteristics and survival. Resuscitation. 2021;158:157-165. doi:10.1016/j.resuscitation.2020.11.016 33221361

[41] Mandigers L, Termorshuizen F, de Keizer NF, . A nationwide overview of 1-year mortality in cardiac arrest patients admitted to intensive care units in the Netherlands between 2010 and 2016. Resuscitation. 2020;147:88-94. doi:10.1016/j.resuscitation.2019.12.029 31926259

[42] Schnaubelt S, Mayr FB, Losert H, . Very long-term survivors of in-hospital and out-of-hospital cardiac arrest show considerable impairment of daily life. Resuscitation. 2022;173:192-200. doi:10.1016/j.resuscitation.2022.01.023 35131405

[43] Allencherril J, Lee PYK, Khan K, Loya A, Pally A. Etiologies of in-hospital cardiac arrest: a systematic review and meta-analysis. Resuscitation. 2022;175:88-95. doi:10.1016/j.resuscitation.2022.03.005 35278525

