# Supplementary material for: Neurologic Recovery at Discharge and Long-Term Survival After Cardiac Arrest
Source: JAMA Netw Open. 2024 Oct 11;7(10):e2439196. doi: 10.1001/jamanetworkopen.2024.39196 (PMC11581594; doi:10.1001/jamanetworkopen.2024.39196)
Supplement: Supplement 1. — eTable 1. Baseline Characteristics of the OHCA Population eTable 2. Baseline Characteristics of the IHCA Population eTable 3. Baseline Characteristics of Eligible Patients With Missing CPC-Score at Discharge eTable 4. Associations Between CPC Score and Long-Term Survival in the Subpopulations With and Without a Diagnosis of IHD, With Shockable and Nonshockable Rhythms and Witnessed Cardiac Arrests With Shockable Rhythm eFigure 1. Adjusted Survival Proportions by Cerebral Performance Category (CPC) for Patients Aged ≤65 Years and >65 Years eFigure 2. Kaplan-Meier Plot for All Patients eFigure 3. Kaplan-Meier Plot for the Subgroups of Patients With Out-of-Hospital Cardiac Arrest (OHCA) and In-Hospital Cardiac Arrest (IHCA) [file jamanetwopen-e2439196-s001.pdf]

Supplemental Online Content

Dillenbeck E, Svensson L, Rawshani A, et al. Neurologic recovery at discharge and long-term survival after cardiac arrest. *JAMA Netw Open*. 2024;7(10):e2439196. doi:10.1001/jamanetworkopen.2024.39196

eTable 1. Baseline Characteristics of the OHCA Population

eTable 2. Baseline Characteristics of the IHCA Population

eTable 3. Baseline Characteristics of Eligible Patients With Missing CPC-Score at Discharge

eTable 4. Associations Between CPC Score and Long-Term Survival in the Subpopulations With and Without a Diagnosis of IHD, With Shockable and Nonshockable Rhythms and Witnessed Cardiac Arrests With Shockable Rhythm

eFigure 1. Adjusted Survival Proportions by Cerebral Performance Category (CPC) for Patients Aged ≤65 Years and >65 Years

eFigure 2. Kaplan-Meier Plot for All Patients

eFigure 3. Kaplan-Meier Plot for the Subgroups of Patients With Out-of-Hospital Cardiac Arrest (OHCA) and In-Hospital Cardiac Arrest (IHCA)

This supplemental material has been provided by the authors to give readers additional information about their work.

© 2024 Dillenbeck E et al. *JAMA Network Open.*

eTable 1: Baseline characteristics of the OHCA population

Characteristic	CPC 1<br>n = 2,914	CPC 2<br>n = 614	CPC 3-4<br>n = 280	SMD<br>CPC 1 vs. CPC 2	SMD<br>CPC 1 vs. CPC 3-4	Missing<br>%
Sex, n(%)				0.08 (-0.01, 0.16)	0.02 (-0.10, 0.14)	<0.1
Female	665 (22.8)	160 (26.1)	66 (23.7)			
Male	2248 (77.2)	454 (73.9)	213 (76.3)			
Age, y, median (IQR)	65 (54.0, 73)	68 (58.3, 77)	65 (52.8, 74)	-0.26 (-0.34, -0.17)	0.00 (-0.12, 0.12)	0.0
Witnessed CA, n(%)	2602 (90.5)	536 (88.7)	236 (85.8)	0.06 (-0.03, 0.14)	0.14 (0.02, 0.27)	1.4
Bystander CPR, n(%)	1575 (56.0)	384 (63.9)	177 (64.8)	-0.16 (-0.25, -0.07)	-0.18 (-0.31, -0.06)	3.2
Shockable rhythm[a], n(%)	2031 (74.3)	412 (71.3)	151 (57.2)	0.07 (-0.02, 0.16)	0.37 (0.24, 0.49)	6.1
Location of CA, n (%)				0.25 (0.16, 0.34)	0.34 (0.22, 0.46)	<0.1
Home	1193 (41.0)	304 (49.5)	159 (57.0)			
Public place	1019 (35.0)	220 (35.8)	79 (28.3)			
Other	700 (24.0)	90 (14.7)	41 (14.7)			
PCI[b], n (%)	635 (47.6)	139 (52.9)	66 (52.0)	-0.11 (-0.24, 0.03)	-0.09 (-0.27, 0.09)	55
CABG[b], n (%)	51 (3.8)	9 (3.5)	5 (4.0)	0.02 (-0.12, 0.15)	-0.01 (-0.19, 0.18)	55
ICD[b], n (%)	444 (25.0)	105 (28.9)	44 (27.5)	-0.09 (-0.20, 0.02)	-0.06 (-0.22, 0.10)	40
TTM (32-26°C)[b], (%)	774 (27.5)	252 (42.9)	155 (58.3)	-0.32 (-0.41, -0.24)	-0.65 (-0.78, -0.53)	3.8
Birthregion, n (%)				0.02 (-0.06, 0.11)	0.04 (-0.09, 0.17)	3.3
Europe	81 (2.9)	17 (2.8)	6 (2.3)			
Nordic countries[c]	2597 (92.3)	558 (92.8)	247 (92.9)			
Other	136 (4.8)	26 (4.3)	13 (4.9)			
Income[d], n (%)				0.14 (0.06, 0.23)	0.12 (0.00, 0.25)	0.2
Q1	618 (21.3)	133 (21.7)	69 (24.7)			
Q2	611 (21.0)	160 (26.1)	62 (22.2)			
Q3	766 (26.4)	159 (25.9)	75 (26.9)			
Q4	912 (31.4)	161 (26.3)	73 (26.2)			
Educational level, n(%)				0.13 (0.04, 0.22)	0.18 (0.05, 0.30)	0.2
Primary	904 (31.1)	221 (36.1)	99 (35.5)			
Secondary	1265 (43.5)	261 (42.6)	102 (36.6)			
Post- secondary ≤2y	336 (11.6)	53 (8.6)	28 (10.0)			
Post- secondary ≤3y	402 (13.8)	78 (12.7)	50 (17.9)			

© 2024 Dillenbeck E et al. *JAMA Network Open.*

Characteristic	CPC 1<br>n = 2,914	CPC 2<br>n = 614	CPC 3-4<br>n = 280	SMD<br>CPC 1 vs. CPC 2	SMD<br>CPC 1 vs. CPC 3-4	Missing<br>%
Comorbidities[e], n(%)						
IHD	948 (32.5)	217 (35.3)	78 (27.9)	-0.06 (-0.15, 0.03)	0.10 (-0.02, 0.22)	0.0
Heart failure	457 (15.7)	131 (21.3)	56 (20.0)	-0.15 (-0.23, -0.06)	-0.11 (-0.24, 0.01)	0.0
COPD	153 (5.3)	33 (5.4)	18 (6.4)	-0.01 (-0.09, 0.08)	-0.05 (-0.17, 0.07)	0.0
Cerebral infarction	151 (5.2)	58 (9.4)	27 (9.6)	-0.16 (-0.25, -0.08)	-0.17 (-0.29, -0.05)	0.0
Diabetes mellitus	355 (12.2)	91 (14.8)	30 (10.7)	-0.08 (-0.16, 0.01)	0.05 (-0.08, 0.17)	0.0
Hypertension	970 (33.3)	244 (39.7)	103 (36.8)	-0.13 (-0.22, -0.05)	-0.07 (-0.20, 0.05)	0.0
Cancer	388 (13.3)	109 (17.8)	41 (14.6)	-0.12 (-0.21, -0.04)	-0.04 (-0.16, 0.08)	0.0
Renal failure	127 (4.4)	44 (7.2)	14 (5.0)	-0.12 (-0.21, -0.03)	-0.03 (-0.15, 0.09)	0.0
Dementia	48 (1.6)	20 (3.3)	23 (8.2)	-0.10 (-0.19, -0.02)	-0.31 (-0.43, -0.18)	0.0

Abbreviations: CA, cardiac arrest; CABG; coronary artery bypass graft; COPD, chronic obstructive pulmonary disease; CPC, cerebral performance category; CPR, cardiopulmonary resuscitation; IQR, interquartile range; ICD, implantable cardioverter defibrillator; IHD, ischemic heart disease; PCI, percutaneous coronary intervention; SMD, standardized mean difference; TTM, targeted temperature management.
[a]Ventricular fibrillation or pulseless ventricular tachycardia
[b]During hospitalization for cardiac arrest
[c]Nordic countries include Sweden, Norway, Denmark and Iceland
[d]Mean disposable income 10 years before cardiac arrest, quartiles
[e]Diagnosed up to 30 days after cardiac arrest

© 2024 Dillenbeck E et al. *JAMA Network Open.*

eTable 2: Baseline characteristics of the IHCA population

Characteristic	CPC 1 n = 4,460	CPC 2 n = 744	CPC 3-4 n = 378	SMD CPC 1 vs. CPC 2	SMD CPC 1 vs. CPC 3-4	Missing %
Sex, n (%)				0.07 (0.00, 0.15	0.09 (-0.02, 0.19)	0.0
Female	1,527 (34.2)	281 (37.8)	145 (38.4)			
Male	2,933 (65.8)	463 (62.2)	233 (61.6)			
Age, y, median (IQR)	70 (60.0, 78)	75 (66.0, 82)	72 (60.3, 81)	-0.36 (-0.44, -0.28)	-0.04 (-0.14, 0.07)	0.0
Witnessed CA, n (%)	4,159 (94.5)	678 (92.7)	342 (91.9)	0.07 (-0.01, 0.15)	0.10 (0.00, 0.21)	1.4
CPR before RRT, n(%)	3,317 (90.3)	600 (92.6)	314 (93.7)	-0.08 (-0.17, 0.00)	-0.13 (-0.24, -0.02)	17
Shockable rhythm[a], n(%)	2,245 (62.6)	276 (49.1)	99 (37.9)	0.27 (0.19, 0.36)	0.51 (0.38, 0.64)	21
Location of CA, n(%)				0.49 (0.41, 0.57)	0.62 (0.51, 0.72)	0.0
Cath lab	955 (21.4)	58 (7.8)	14 (3.7)			
ER	550 (12.3)	94 (12.6)	59 (15.6)			
General ward	1,060 (23.8)	302 (40.6)	153 (40.5)			
Intensive care	1,335 (29.9)	222 (29.8)	109 (28.8)			
Other	560 (12.6)	68 (9.1)	43 (11.4)			
PCI[b], n (%)	505 (32.7)	50 (17.8)	8 (5.2)	0.35 (0.22, 0.48)	0.75 (0.58, 0.92)	65
CABG[b], n (%)	32 (2.1)	6 (2.1)	0 (0.0)	0.00 (-0.13, 0.12)	0.21 (0.04, 0.37)	65
ICD[b], n (%)	75 (7.9)	18 (10.0)	2 (1.9)	-0.07 (-0.23, 0.09)	0.28 (0.08, 0.48)	78
TTM (32-26°C)[b], (%)	229 (5.2)	74 (10.2)	50 (13.6)	-0.19 (-0.27, -0.11)	-0.29 (-0.40, -0.18)	1.4
Birthregion, n (%)				0.10 (0.02, 0.18)	0.05 (-0.06, 0.15)	3.8
Europe	122 (2.8)	11 (1.5)	12 (3.3)			
Nordic countries[c]	3,962 (92.2)	673 (94.5)	330 (90.9)			
Other	213 (5.0)	28 (3.9)	21 (5.8)			
Income[d], n (%)				0.19 (0.11, 0.26)	0.28 (0.17, 0.39)	0.6
Q1	1,106 (24.9)	200 (27.1)	116 (31.4)			
Q2	1,152 (26.0)	233 (31.5)	117 (31.6)			
Q3	1,100 (24.8)	176 (23.8)	83 (22.4)			
Q4	1,079 (24.3)	130 (17.6)	54 (14.6)			
Educational level, n (%)				0.08 (0.01, 0.16)	0.10 (-0.01, 0.21)	0.7
Primary	1,566 (35.3)	273 (37.0)	144 (38.9)			
Secondary	1,826 (41.2)	304 (41.2)	144 (38.9)			
Post-secondary ≤2y	439 (9.9)	56 (7.6)	29 (7.8)			

Characteristic	CPC 1 n = 4,460	CPC 2 n = 744	CPC 3-4 n = 378	SMD CPC 1 vs. CPC 2	SMD CPC 1 vs. CPC 3-4	Missing %
Post-secondary ≤3y	606 (13.7)	105 (14.2)	53 (14.3)			
Comorbidities[e], n (%)						
IHD	1,921 (43.1)	301 (40.5)	120 (31.7)	0.05 (-0.02, 0.13)	0.24 (0.13, 0.34)	0.0
Heart failure	978 (21.9)	232 (31.2)	79 (20.9)	-0.21 (-0.29, -0.13)	0.03 (-0.08, 0.13)	0.0
COPD	362 (8.1)	80 (10.8)	32 (8.5)	-0.09 (-0.17, -0.01)	-0.01 (-0.12, 0.09)	0.0
Cerebral infarction	306 (6.9)	139 (18.7)	73 (19.3)	-0.36 (-0.44, -0.28)	-0.38 (-0.48, -0.27)	0.0
Diabetes mellitus	944 (21.2)	204 (27.4)	93 (24.6)	-0.15 (-0.22, -0.07)	-0.08 (-0.19, 0.02)	0.0
Hypertension	2,204 (49.4)	445 (59.8)	202 (53.4)	-0.21 (-0.29, -0.13)	-0.08 (-0.19, 0.02)	0.0
Cancer	983 (22.0)	191 (25.7)	79 (20.9)	-0.09 (-0.16, -0.01)	0.03 (-0.08, 0.13)	0.0
Renal failure	457 (10.2)	108 (14.5)	51 (13.5)	-0.13 (-0.21, -0.05)	-0.10 (-0.21, 0.00)	0.0
Dementia	78 (1.7)	75 (10.1)	45 (11.9)	-0.36 (-0.44, -0.28)	-0.41 (-0.52, -0.31)	0.0

Abbreviations: CA, cardiac arrest; CABG; coronary artery bypass graft; COPD, chronic obstructive pulmonary disease; CPC, cerebral performance category; CPR, cardiopulmonary resuscitation; ER, emergency room, ICD, implantable cardioverter defibrillator; IQR, interquartile range; IHD, ischemic heart disease; PCI, percutaneous coronary intervention; RRT, rapid response team; SMD, standardized mean difference; TTM, targeted temperature management. [a]Ventricular fibrillation or pulseless ventricular tachycardia

[b]During hospitalization for cardiac arrest

[c]Nordic countries include Sweden, Norway, Denmark and Iceland

[d]Mean disposable income 10 years before cardiac arrest, quartiles

[e]Diagnosed up to 30 days after cardiac arrest

© 2024 Dillenbeck E et al. *JAMA Network Open.*

eTable3: Baseline characteristics of eligible patients with missing CPC-score at discharge

Characteristic	n = 2657	Missing %
OHCA, n (%)	1353 (50.9)	0.0
IHCA, n (%)	1304 (49.1)	0.0
Male, n (%)	1819 (68.5)	0.0
Female, n (%)	838 (31.7)	0.0
Age, y, median (IQR)	69 (58.0, 77)	0.0
Witnessed CA, n (%)	2300 (88.1)	1.7
Shockable rhythm[a], n(%)	1203 (55.4)	18
PCI[b], n (%)	379 (37.7)	62
CABG[b], n (%)	26 (2.6)	62
ICD[b], n (%)	202 (20.6)	63
TTM (32-26°C)[b], (%)	293 (18.7)	41
Birthregion, n (%)		4.5
Europe	67 (2.6)	
Nordic countries[c]	2348 (92.7)	
Other	119 (4.7)	
Income[d], n (%)		4.3
Q1	731 (28.7)	
Q2	637 (25.0)	
Q3	613 (24.1)	
Q4	563 (22.1)	
Educational level, n (%)		4.3
Primary	928 (36.5)	
Secondary	1060 (41.7)	
Post- secondary ≤2y	247 (9.7)	
Post- secondary ≤3y	309 (12.1)	
Comorbidities[e]		
IHD, n (%)	920 (34.6)	0.0
Heart failure, n (%)	542 (20.4)	0.0
COPD, n (%)	217 (8.2)	0.0
Cerebral infarction, n(%)	245 (9.2)	0.0
Diabetes mellitus, n(%)	497(18.7)	0.0
Hypertension, n (%)	1153 (43.4)	0.0
Cancer, n (%)	474 (17.8)	0.0
Renal failure, n(%)	269 (10.1)	0.0
Dementia, n (%)	109 (4.1)	0.0

Abbreviations: CA, cardiac arrest; CABG; coronary artery bypass graft; COPD, chronic obstructive pulmonary disease; CPC, cerebral performance category; IQR, interquartile range; ICD, implantable cardioverter defibrillator; IHD, ischemic heart disease; PCI, percutaneous coronary intervention; SMD, standardized mean difference; TTM, targeted temperature management.
[a]Ventricular fibrillation or pulseless ventricular tachycardia
[b]During hospitalization for cardiac arrest
[c]Nordic countries include Sweden, Norway, Denmark and Iceland
[d]Mean disposable income 10 years before cardiac arrest, quartiles
[e]Diagnosed up to 30 days after cardiac arrest

© 2024 Dillenbeck E et al. *JAMA Network Open.*

eTable4: Associations between CPC score and long-term survival in the subpopulations with and without a diagnosis of IHD, with shockable and non-shockable rhythms and witnessed cardiac arrests with shockable rhythm.

	N	Event N	Adjusted HR (95% CI)	p-value
IHD				
CPC 1			—	
CPC 2			1.58 (1.34-1.86)	<0.001
CPC 3-4			2.18 (1.73- 2.74)	<0.001
No IHD				
CPC 1	3203	684	—	
CPC 2	615	215	1.53 (1.30- 1.79)	<0.001
CPC 3-4	308	141	2.58 (2.13-3.12)	<0.001
Non-shockable rhythm				
CPC 1	1714	621	—	
CPC 2	387	184	1.48 (1.25-1.75)	<0.001
CPC 3-4	228	118	2.19 (1.78- 2.69)	<0.001
Shockable rhythm				
CPC 1	3523	815	—	
CPC 2	603	228	1.56 (1.34- 1.82)	<0.001
CPC 3-4	217	107	2.61 (2.12- 3.21)	<0.001
Witnessed and shockable rhythm				
CPC 1	3320	768	—	
CPC 2	553	217	1.59 (1.36-1.86)	<0.001
CPC 3-4	200	100	2.64 (2.13-3.27)	<0.001

Abbreviations: CI, confidence interval; CPC, cerebral performance category, IHD, ischemic heart disease; HR, hazard ratio

© 2024 Dillenbeck E et al. *JAMA Network Open.*

eFigure1: Adjusted survival proportions by cerebral performance category (CPC) for patients aged ≤65 years and >65 years.

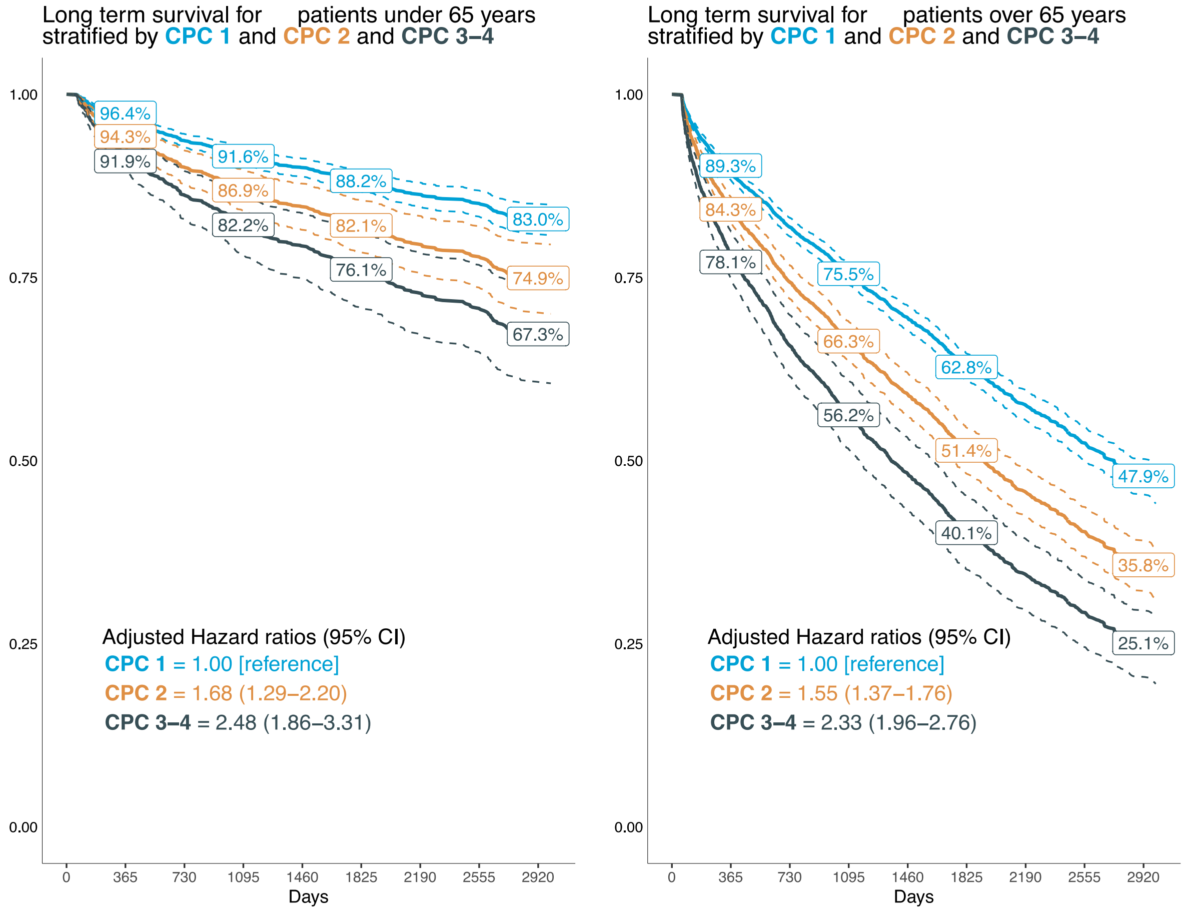

Adjusted survival proportions by CPC using multivariable Cox proportional hazards regression models, adjusted for age, sex, initial rhythm, witnessed cardiac arrest, bystander CPR, year of cardiac arrest, OHCA/IHCA, birth region, income and comorbidities (ischemic heart disease, heart failure, chronic obstructive pulmonary disease, diabetes mellitus, cancer, renal failure and dementia). Adjusted survival proportions at 1, 3, 5 and 8 years of follow-up are shown as percentages on the curves. CI indicates confidence interval; CPC, cerebral performance category.

© 2024 Dillenbeck E et al. *JAMA Network Open.*

eFigure2: **Kaplan-Meier plot for all patients.**

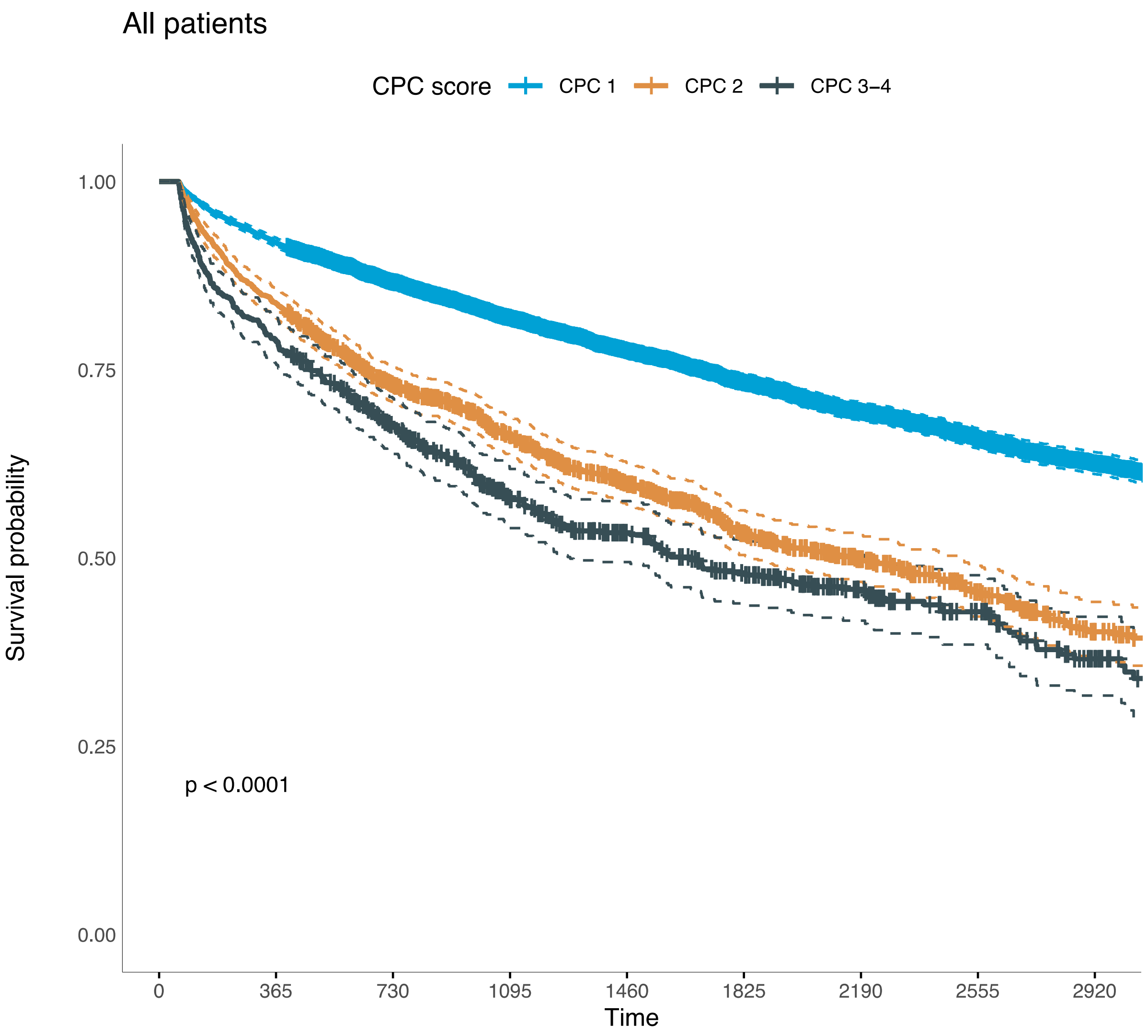

Numbers at risk

CPC score	0	365	730	1095	1460	1825	2190	2555	2920
CPC 1	7374	6778	5763	4775	3900	3091	2377	1773	1242
CPC 2	1358	1139	898	712	557	400	272	184	108
CPC 3–4	658	519	399	291	219	173	120	84	50

Time

CPC indicates cerebral performance category

© 2024 Dillenbeck E et al. *JAMA Network Open.*

eFigure3: **Kaplan-Meier plot for the subgroups of patients with out-of-hospital cardiac arrest (OHCA) and in-hospital cardiac arrest (IHCA).**

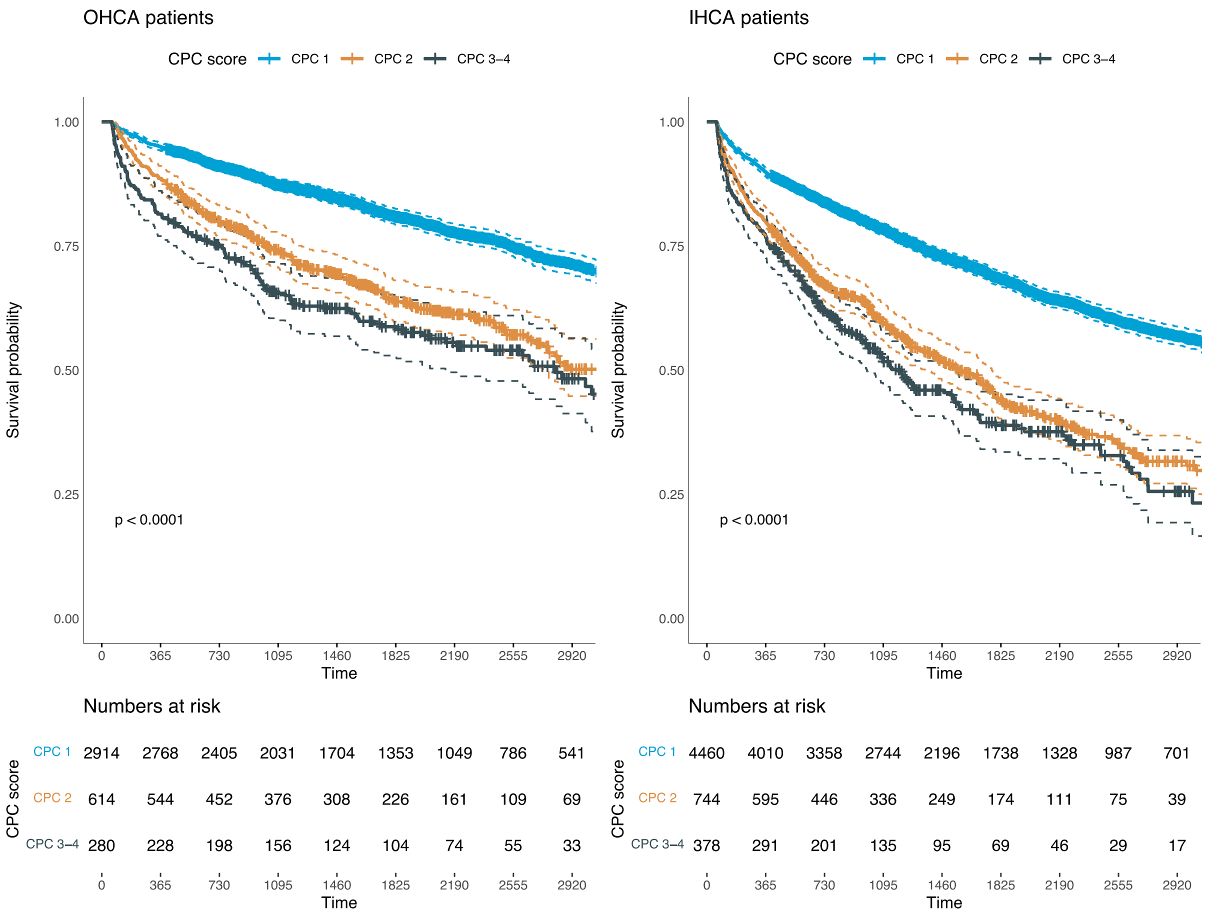

CPC indicates cerebral performance category.

© 2024 Dillenbeck E et al. *JAMA Network Open.*
