# Supplementary material for: Neurologic Recovery at Discharge and Long-Term Survival After Cardiac Arrest
Source: JAMA Netw Open. 2024 Oct 11;7(10):e2439196. doi: 10.1001/jamanetworkopen.2024.39196 (PMC11581594; doi:10.1001/jamanetworkopen.2024.39196)
Supplement: Supplement 2. — Data Sharing Statement [file jamanetwopen-e2439196-s002.pdf]

Data Sharing Statement

Dillenbeck. Neurologic Recovery at Discharge and Long-Term Survival After Cardiac Arrest. *JAMA Netw Open*. Published October 11, 2024. doi:10.1001/jamanetworkopen.2024.39196

Data
Data available: No

Additional Information
Explanation for why data not available: Data cannot be shared due to data protection requirements within national law.
